# Cardiac Tamponade and Arrest Secondary to Simultaneous Gastric and Sigmoid Volvulus With Sigmoid Obstruction From an Incarcerated Left Inguinal Hernia

**DOI:** 10.7759/cureus.103017

**Published:** 2026-02-05

**Authors:** Bella Vogt, Kabir A Torres, Hongyi Cui

**Affiliations:** 1 Department of Surgery, University of Massachusetts Chan Medical School, Worcester, USA

**Keywords:** acute care surgery and gastrointestinal surgery, cardiac tamponade, mesentero-axial gastric volvulus, pea arrest, sigmoid volvulus, strangulated inguinal hernia

## Abstract

Sigmoid volvulus is a well-documented cause of large bowel obstruction, often occurring in elderly or neurologically impaired individuals. While typically straightforward in diagnosis, concurrent anatomical abnormalities can obscure the clinical presentation and lead to catastrophic outcomes. This report presents an exceptionally rare case of simultaneous gastric and sigmoid volvulus with incarceration of the sigmoid colon within a left inguinal hernia, resulting in cardiac tamponade-like physiology and cardiac arrest.

A 78-year-old man with cognitive impairment and bilateral inguinal hernias was found in pulseless electrical activity (PEA) arrest at his group home. Return of spontaneous circulation (ROSC) was achieved twice following cardiopulmonary resuscitation. On arrival at the emergency department, the patient was profoundly unstable, with a markedly distended abdomen and tense bilateral hernias. Computed tomography revealed a distended sigmoid colon consistent with volvulus, with the proximal sigmoid incarcerated within the left inguinal hernia. The entire stomach was also herniated into the thoracic cavity, causing severe left atrial compression. Laboratory results showed a serum lactate level of 18 mmol/L, consistent with global tissue hypoperfusion. Emergent exploratory laparotomy revealed a torsed, ischemic sigmoid colon and gastric volvulus with mechanical cardiac compression. Surgical management included reduction of the sigmoid and hiatal hernias, sigmoid colectomy with end colostomy, and anterior gastropexy. Despite aggressive resuscitation, the patient developed refractory metabolic acidosis and multiorgan failure, culminating in death on postoperative day one.

This case demonstrates a rare and lethal confluence of gastrointestinal and cardiovascular pathophysiology. The sigmoid volvulus and gastric herniation produced both obstructive and compressive hemodynamic compromise, culminating in circulatory collapse. The patient’s outcome was further worsened by the physiologic insult of preceding cardiac arrests and the systemic inflammatory state characteristic of post-cardiac arrest syndrome.

This case highlights the importance of early imaging and a high index of clinical suspicion for complex anatomic causes of obstructive shock. Simultaneous gastric and sigmoid volvulus with herniation can produce catastrophic hemodynamic consequences, including cardiac tamponade physiology. Multidisciplinary coordination and rapid intervention are essential, although prognosis remains poor in patients presenting with severe ischemia and post-resuscitation instability.

## Introduction

Sigmoid volvulus is a well-recognized yet potentially fatal cause of large bowel obstruction, particularly in elderly or neurologically impaired individuals [1,2]. The condition is characterized by torsion of a redundant sigmoid colon around its mesenteric axis, resulting in luminal obstruction and possible vascular compromise. The classical presentation is a triad of abdominal pain, abdominal distention, and obstipation. While most cases present in a relatively straightforward fashion, anatomical complexity and multiple concurrent hernias can obscure diagnosis and complicate management. This case highlights a rare presentation of sigmoid volvulus with simultaneous inguinoscrotal and hiatal herniation, culminating in multisystem organ failure and death.

## Case presentation

A 78-year-old male with a known cognitive disability and bilateral inguinal hernias was discovered in pulseless electrical activity (PEA) arrest at his group home while eating breakfast. Emergency medical services initiated cardiopulmonary resuscitation (CPR), and return of spontaneous circulation (ROSC) was achieved in the field. On arrival in the emergency department, he experienced a second PEA arrest, requiring additional CPR before ROSC was restored. Initial laboratory findings are shown in Table 1. On physical examination, the abdomen was markedly distended, and both inguinal hernias were tense and prominent.

**Table 1:** Initial Laboratory Findings mmol/L: millimoles per liter; mg/dL: milligrams per deciliter; mL/min/1.73 m²: milliliters per minute per 1.73 square meters of body surface area; U/L: units per liter; 10^3^/L: cells per liter; g/dL: grams per liter; mm Hg: millimeters of mercury.

Parameter	Patient Value	Reference Range
Sodium (mmol/L)	146	135–145
Potassium (mmol/L)	3.6	3.5–5.3
CO_2_ (mmol/L)	7	22–32
Anion gap	41	5–15
Glucose (mg/dL)	190	65–99
Urea (mg/dL)	22	7–23
Creatinine (mg/dL)	1.8	0.60–1.30
Estimated glomerular filtration rate (mL/min/1.73m^2^)	38	>60
Lactic acid (mmol/L)	18.6	0.5–1.9
AST (U/L)	338	10–40
ALT (U/L)	333	10–40
Total bilirubin (mg/dL)	1.6	0.2–1.2
WBC (×10^3^/uL)	26.1	3.8–10.8
Hemoglobin (g/dL)	15.6	13.2–17.1
Hematocrit (%)	54.3	38.5–50.0
INR	1.3	0.9–1.1
Protime (seconds)	14.2	9.6–12.4
Venous pH	<7.00	7.31–7.41
Venous pCO2 (mm Hg)	114	41–51
Venous pO2 (mm Hg)	46	35–40

**Figure 1:**
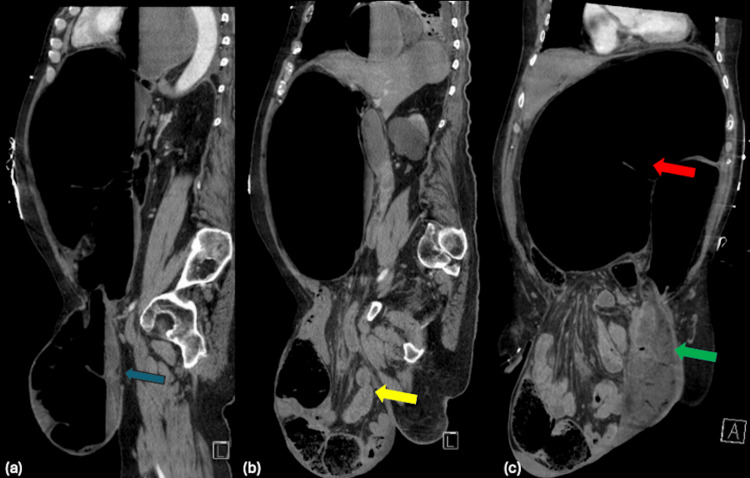
(a) Sagittal view of left inguinoscrotal hernia containing dilated sigmoid colon (blue arrow). (b) Sagittal view of right inguinoscrotal hernia containing a majority of small bowel and mesentery (yellow arrow). (c) Coronal view of right inguinoscrotal hernia (green arrow). Dilated, gas-filled sigmoid colon noted proximally in the peritoneal cavity (red arrow).

A computed tomography (CT) scan of the abdomen and pelvis revealed a markedly distended sigmoid colon consistent with sigmoid volvulus, with a short segment of the proximal sigmoid incarcerated within the left inguinal hernia (Figure 1a-c).

**Figure 2:**
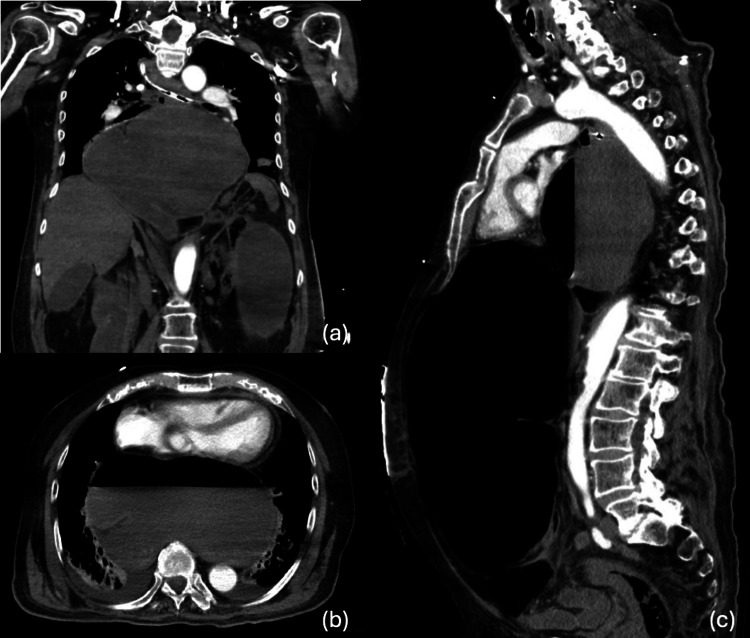
(a) Hiatal hernia with mesoaxial gastric volvulus. (b) Axial view of gastric herniation causing cardiac compression. (c) Sagittal view of sigmoid distention due to sigmoid volvulus.

Additionally, the entire stomach was herniated above the diaphragm and massively distended, causing a significant mass effect on the mediastinum with severe compression of the left atrium (Figures 2a-c). There was also diffuse bowel distension, with long segments of bowel extending into large bilateral inguinal hernias. Associated mesenteric congestion and intra-hernia fluid raised concern for bowel ischemia.

This constellation of findings supported the clinical suspicion for bowel ischemia and physiologic cardiac tamponade contributing to the patient’s hemodynamic instability. Preoperative laboratory values revealed a critically elevated serum lactate level of 18 mmol/L, consistent with severe tissue hypoperfusion and possible bowel ischemia. Discussions were held with the patient’s healthcare proxy (HCP), and operative intervention was offered with the understanding that the patient carried a significant perioperative risk of morbidity given his two-fold PEA arrest. The decision was made to proceed with aggressive measures and operative intervention per the HCP’s wishes, despite the known grim prognosis.

**Figure 3:**
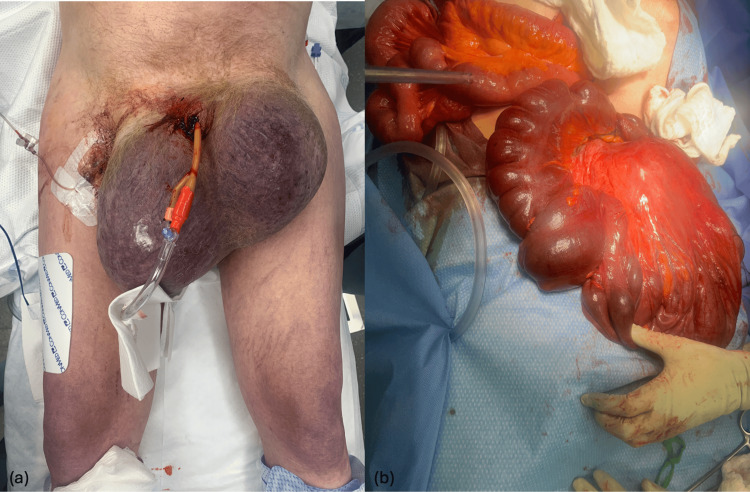
(a) Gross view of large bilateral inguinoscrotal hernia. (b) Severely dilated sigmoid colon after reduction from inguinoscrotal hernia.

The patient underwent emergent exploratory laparotomy. Given that the patient was found to be in extremis, the operative goal was damage control. Intraoperative findings included a torsed, ischemic sigmoid colon, which was surgically reduced (Figures 3a-b). The sigmoid colon was reduced from the left inguinal hernia, and an enterotomy was created in a segment of colon planned for resection to decompress the significantly dilated bowel. The hiatal hernia was also addressed with expeditious reduction of the stomach from the thoracic cavity to relieve the cardiac tamponade caused by mass effect, along with reduction of the mesoaxial volvulus and anterior gastropexy. A sigmoid colectomy with end colostomy creation was performed. An abdominal washout was conducted, and a negative pressure dressing was placed with a plan for return to the operating room in 24 hours. Intraoperatively, the patient produced no urine output, suggestive of acute renal failure. Postoperatively, he was transferred to the intensive care unit (ICU) in critical condition, requiring multiple vasopressors for circulatory support. Despite aggressive resuscitative measures, he remained oliguric and severely acidotic, with a worsening base deficit despite attempts at correction.

On postoperative day one, the patient suffered a final cardiac arrest. Resuscitation efforts were unsuccessful. The presumed cause of death was profound metabolic derangement due to multiorgan failure, including ischemic colitis, acute kidney injury, and mechanical tamponade with left atrial compression secondary to hiatal hernia mass effect.

## Discussion

Sigmoid volvulus is the most frequent form of colonic volvulus and represents 10-15% of large bowel obstructions in the Western world, with higher rates in endemic regions such as Africa, South America, and the Middle East. Risk factors include advanced age, cognitive impairment, institutionalization, chronic constipation, and anatomic abnormalities such as dolichosigmoid [1,3].

This case exemplifies how multiple coexisting anatomic abnormalities can interact to create a lethal physiologic scenario. The sigmoid volvulus, likely precipitated by underlying colonic redundancy and constipation, was complicated by herniation into the inguinoscrotal sac. Simultaneously, the presence of a large paraesophageal hernia containing the entire stomach resulted in mechanical compression of the left atrium, producing a tamponade-like state and exacerbating the patient’s shock. This dual obstruction, gastrointestinal and cardiovascular, resulted in profound systemic hypoperfusion, as evidenced by the patient’s markedly elevated lactate level and intraoperative anuria.

Although prompt surgical intervention was undertaken, the patient’s critical preoperative state and persistent metabolic acidosis portended a poor prognosis. Recent studies highlight that delayed presentation and advanced age significantly increase mortality in cases of sigmoid volvulus [4,5].

Perioperative cardiac arrest, though uncommon, remains a devastating complication associated with significant morbidity and mortality. Over the past decade, evidence has consistently shown that cardiac arrest triggers a profound systemic inflammatory response marked by global ischemia-reperfusion injury and myocardial dysfunction [6]. Even in the absence of obstructive coronary disease, many patients develop post-arrest myocardial stunning, characterized by depressed cardiac output, hypotension, and increased vasopressor requirements [7]. The ensuing systemic inflammatory cascade further amplifies endothelial injury and microcirculatory derangements, predisposing patients to perioperative complications such as acute kidney injury, coagulopathy, and progressive metabolic acidosis [8].

Moreover, data from large registries and multicenter cohorts demonstrate that survival to hospital discharge after perioperative cardiac arrest is particularly poor among patients undergoing emergency or complex procedures, especially those of advanced age, with substantial comorbidities, or with non-shockable presenting rhythms such as pulseless electrical activity [9-11].

In the case presented, the patient’s two episodes of pulseless electrical activity arrest prior to surgery, coupled with evidence of profound systemic hypoperfusion, including markedly elevated serum lactate and intraoperative anuria, placed him at exceedingly high risk for poor surgical outcome. Despite prompt operative intervention, his persistent metabolic derangement and circulatory failure ultimately culminated in death, consistent with the grim prognosis reported in the literature for surgical candidates following recent cardiac arrest.

## Conclusions

This case illustrates the complex interplay of abdominal and thoracic pathology in an elderly patient with sigmoid volvulus. It underscores the importance of early recognition and imaging in atypical presentations and reinforces the need for swift, multidisciplinary management in patients presenting with signs of obstructive shock and anatomic derangements. Despite timely surgical intervention, the patient succumbed to multiorgan failure due to the severity of the initial ischemia and cardiovascular compromise.
